# Clinical classification, visual outcomes, and optical coherence tomographic features of 48 patients with posterior sympathetic ophthalmia

**DOI:** 10.1186/s13023-022-02258-0

**Published:** 2022-03-04

**Authors:** Hong Zhuang, Rui Zhang, Ting Zhang, Qing Chang, Gezhi Xu

**Affiliations:** 1 Department of Ophthalmology, Eye and ENT Hospital, Fudan University, 83 Fenyang Road, Shanghai, 200031 China; 2 Shanghai Key Laboratory of Visual Impairment and Restoration, and NHC Key Laboratory of Myopia (Fudan University), Shanghai, 200031 China

**Keywords:** Sympathetic ophthalmia, Clinical classification, Visual outcomes, Optical coherence tomography

## Abstract

**Background:**

To investigate the clinical manifestations, visual outcomes and optical coherence tomographic (OCT) features of patients with posterior sympathetic ophthalmia (PSO).

**Methods:**

We performed a retrospective review of 48 patients diagnosed with PSO between January 2013 and December 2019. We compared the clinical and OCT features among different clinical types of PSO.

**Results:**

PSO could be classified into two types according to whether the fundus exhibited serous retinal detachment (SRD) or multifocal choroiditis (MFC). There were 41 patients (85.4%) with SRD and 7 patients (14.6%) with MFC. The latent period of patients with MFC was significantly longer than that of patients with SRD (*P* = 0.002). The final visual acuity of patients with MFC was significantly worse than that of patients with SRD (*P* = 0.0001). In patients with acute SRD, OCT revealed that the mean height of retinal detachment in the fovea was 528.8 ± 437.5 μm. After treatment, the retina reattached in all patients and the band structures of the outer retina were restored in most patients (92.7%). In patients with acute MFC, the OCT images revealed inflammatory lesions on the retinal pigment epithelium layer. After treatment, the OCT images showed hyperreflective fibrosis of the lesions and loss of the outer retinal band structures in all patients.

**Conclusions:**

We found that PSO could be classified according to the presence of SRD or MFC. The visual prognosis differed significantly between these types of PSO. OCT imaging is useful for clinical classification and monitoring of retinal changes after treatment.

**Supplementary Information:**

The online version contains supplementary material available at 10.1186/s13023-022-02258-0.

## Background

Uveitis is a group of vision-threatening intraocular inflammatory diseases [1]. Sympathetic ophthalmia is a rare form of bilateral uveitis that occurs after open globe injury or intraocular surgery to the inciting eye. It is characterized by an autoimmune response to the exposure of ocular tissue antigens. Most patients present with posterior sympathetic ophthalmia (PSO) with mild or no anterior segment inflammation [2]. Serous retinal detachment (SRD) is the most common manifestation of PSO [2, 3]. Some reports have revealed multifocal choroiditis (MFC) is also a manifestation of PSO [4, 5].

It has been estimated that sympathetic ophthalmia occurs in 0.02–0.05% of patients with ocular trauma and in 0.01% of patients undergoing ophthalmic surgery [6]. Because of its rarity, most of the clinical and optical coherence tomography (OCT) studies of sympathetic ophthalmia were case reports or small case-series [7, 8]. Previous OCT studies of small numbers of patients initially revealed SRD with choroidal thickening in the acute stage of PSO [9–11]. However, the clinical and OCT features of PSO are still poorly understood. In this study, we investigated the clinical manifestations, visual prognosis, and OCT features of a large number of patients with PSO.

## Methods

This research adhered to the tenets of the Declaration of Helsinki and was approved by the Ethics Committee of the Eye and ENT Hospital of Fudan University (Shanghai, China). Written informed consent was obtained from each patient.

We performed a retrospective review of patients diagnosed with PSO at the Eye and ENT Hospital between January 2013 and December 2019. Based on the diagnostic criteria proposed by the standardization of uveitis nomenclature working group [6], the diagnosis of sympathetic ophthalmia in our study needs to meet the following conditions: (1) history of open globe injury or intraocular surgery to one eye; (2) bilateral uveitis; if the inciting eye was not viewable (e.g. due to enucleation, phthisis bulbi, opaque cornea), then inflammation is present in the sympathizing eye; (3) ocular infectious diseases, systemic autoimmune diseases, and tumor associated masquerade syndrome were excluded from the differential diagnosis. The histopathologic characteristics of the inciting eye could also be considered to support the diagnosis of sympathetic ophthalmia. The diagnosis of sympathetic ophthalmia was ultimately confirmed by two experienced uveitis specialists (Gezhi Xu and Qing Chang). During the study period, 49 patients were diagnosed with sympathetic ophthalmia at our institution. One patient presented with iridocyclitis and the other 48 patients presented with posterior uveitis (PSO); these 48 patients were included in this study.

The medical records of the 48 patients with PSO were reviewed to collect information on patient demographics, inciting event, latent period, treatments, and best-corrected visual acuity. All patients underwent comprehensive ophthalmic examinations, including slit-lamp biomicroscopy, ocular fundus examination, B-scan ultrasonography, and OCT examination. Fundus fluorescein angiography (FFA) was performed in a part of patients.

All 48 patients received oral systemic corticosteroids in the acute stage at a starting dose of 1–1.5 mg/kg body weight that was then slowly tapered. If the corticosteroids did not completely control the inflammation, the patients were also treated with an immunosuppressive agent (cyclosporine). The total duration of treatment was over 6 months for all patients. At the last follow-up, all patients had discontinued the medical treatment.

OCT images were obtained using the Spectralis spectral-domain OCT instrument (Heidelberg Engineering, Heidelberg, Germany). OCT scans were performed along horizontal and vertical lines through the fovea centralis. Obvious retinal lesions were scanned. After treatment, the eyes were re-scanned using the eye-tracking-based follow-up function in Spectralis OCT to examine the same lesion site and precisely evaluate the retinal changes. If SRD was apparent in the fundus, we manually measured the height of retinal detachment in the fovea. The height of retinal detachment was measured with a built-in caliper from the outer border of the neurosensory retina to the inner border of the retinal pigment epithelium (RPE).

Sympathetic ophthalmia is traditionally defined as bilateral uveitis, but it may be difficult to view inflammation in the inciting eye due to prior enucleation, phthisis bulbi, or corneal opacity. Furthermore, the manifestations of posterior uveitis in the inciting eye can be influenced by fundus complications such as retinal injury and proliferative vitreoretinopathy. Because it was difficult to analyze the clinical features of sympathetic ophthalmia in the inciting eyes, we primarily analyzed the clinical features of the contralateral sympathizing eyes.

### Data analysis

We analyzed the visual acuity and OCT images of the sympathizing eyes in the acute stage and at the last follow-up after treatment. Visual acuity values were converted to the logarithm of the minimal angle of resolution (LogMAR). The patients were divided into two subgroups according to the inciting event or the fundus manifestations. The paired rank sum test was used to compare visual acuity before and after treatment in each subgroup. The nonparametric rank sum test was used to compare continuous data (visual acuity and age) between two subgroups. Because some cells in the contingency tables included fewer than five patients, Fisher’s exact test was used to compare the categorical variables between the two subgroups. Spearman’s correlation coefficient was used to analyze correlations between the final visual acuity and other parameters (the initial visual acuity or age). Spearman’s correlation coefficient was also used to analyze correlations between the retinal detachment height and other parameters (the initial or final visual acuity). A *P* value of < 0.05 was considered statistically significant. The statistical analyses were performed using Stata 11.0 statistical software (Stata Corporation, College Station, TX, USA).

## Results

### Patient characteristics, clinical classification, and visual outcomes

This study included 48 patients with PSO, comprising 42 males and 6 females. The mean ± standard deviation age was 45.8 ± 15.2 years (range 10–73 years, median 47 years). The inciting event was ocular trauma in 39 patients (81%; trauma group) and intraocular surgery in nine patients (19%; surgery group). All 39 cases of ocular trauma involved open globe injury. In the nine patients whose inciting event was intraocular surgery, the procedure was vitrectomy in seven patients, cataract extraction (phacoemulsification with intraocular lens implantation) in one patient, and anti-glaucoma surgery (trabeculectomy combined with a peripheral iridectomy) in one patient. The latent period was < 3 months in 21 patients, 3 months to 1 year in 11 patients, and > 1 year in 16 patients. The follow-up time after the start of treatment was 15.3 ± 8.4 months (range 8–36 months, median 12 months).

As shown in Table 1, there were no significant differences in the sex, age, or latent period between the trauma and surgery groups. Table 2 shows the visual acuity before treatment and at the last follow-up after treatment in the trauma and surgery groups. The final visual acuity was significantly better than the initial visual acuity in the trauma group (*P* = 0.0001) but not in the surgery group (*P* = 0.7669). Although the final visual acuity tended to be better in the trauma group than in the surgery group, this was not statistically significant (*P* = 0.2671).

**Table 1:** Sex, age, and latent period of patients with posterior sympathetic ophthalmia according to the inciting event

Group	Sex (M/F)	Age (years), mean ± SD (median)	Latent period		
			< 3 months	3 months to 1 year	> 1 year
Trauma group (*n* = 39)	34/5	46.6 ± 16.1 (49)	17	9	13
Surgery group (*n* = 9)	8/1	42.4 ± 10.6 (45)	4	2	3
*P*-value	1.000	0.4512	0.998		

*M* male, *F* female, *SD* standard deviation

**Table 2:** Initial and final visual acuity of patients with posterior sympathetic ophthalmia according to the inciting event

	Trauma group (*n* = 39)	Surgery group (*n* = 9)	*P *value
Initial VA (LogMAR) mean ± SD (range)	0.79 ± 0.54 (2.0 to 0.0)	0.71 ± 0.40 (1.3 to 0.2)	0.8213
Final VA (LogMAR) mean ± SD (range)	0.38 ± 0.54 (2.0 to − 0.1)	0.73 ± 0.80 (2.0 to − 0.1)	0.2671
*P*-value	0.0001	0.7669	

*VA* visual acuity, *LogMAR* logarithms of the minimum angle of resolution

**Fig. 1:**
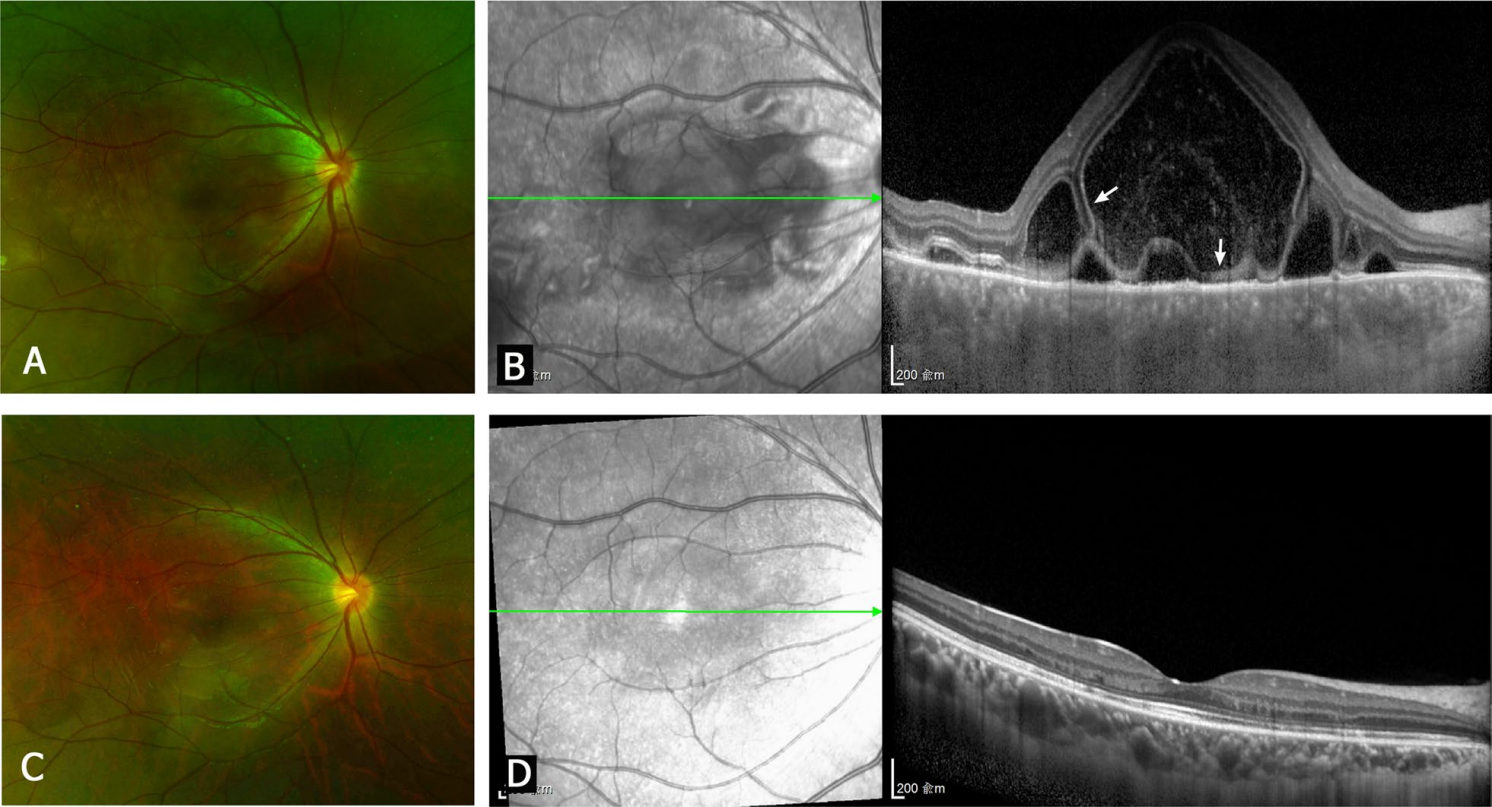
A 20-year-old patient with posterior sympathetic ophthalmia (PSO) accompanied by serous retinal detachment (SRD). Fundus photography and optical coherence tomography (OCT) images of the right eye (sympathizing eye). **a, b** Acute stage. **(a)** The fundus photograph shows SRD in the macular area. **b** The OCT image shows low reflective exudation of amorphous materials in the subretinal space. The arrows indicate the membranous structure. **c, d** Nine months after treatment. **d** The OCT image shows reattachment of the retina and the bands of the external limiting membrane and ellipsoid zone

**Fig. 2:**
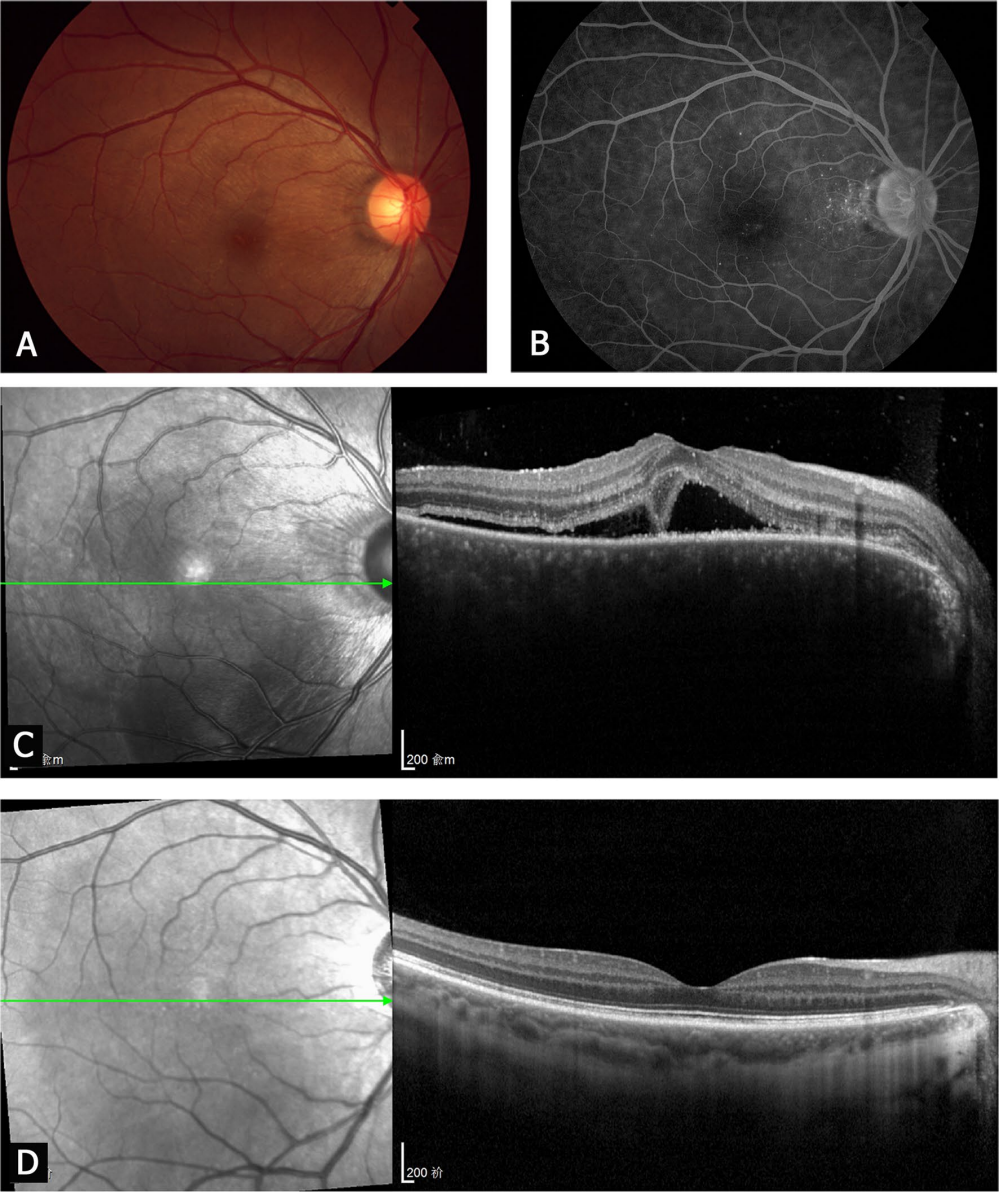
A 31-year-old patient with posterior sympathetic ophthalmia (PSO) accompanied by serous retinal detachment (SRD). Fundus photography, fundus fluorescein angiography (FFA) and optical coherence tomography (OCT) images of the right eye (sympathizing eye). **a-c** Acute stage. **a** The fundus photograph shows retinal edema and choroidal folds. **b** FFA shows hyperfluorescent pinpoint leaks in the posterior pole of fundus. **c** The OCT image shows SRD and the undulating retinal pigment epithelium (RPE). **d** Ten months after treatment. The OCT image shows reattachment of the retina and the bands of the external limiting membrane and ellipsoid zone. The RPE layer has flattened

**Fig. 3:**
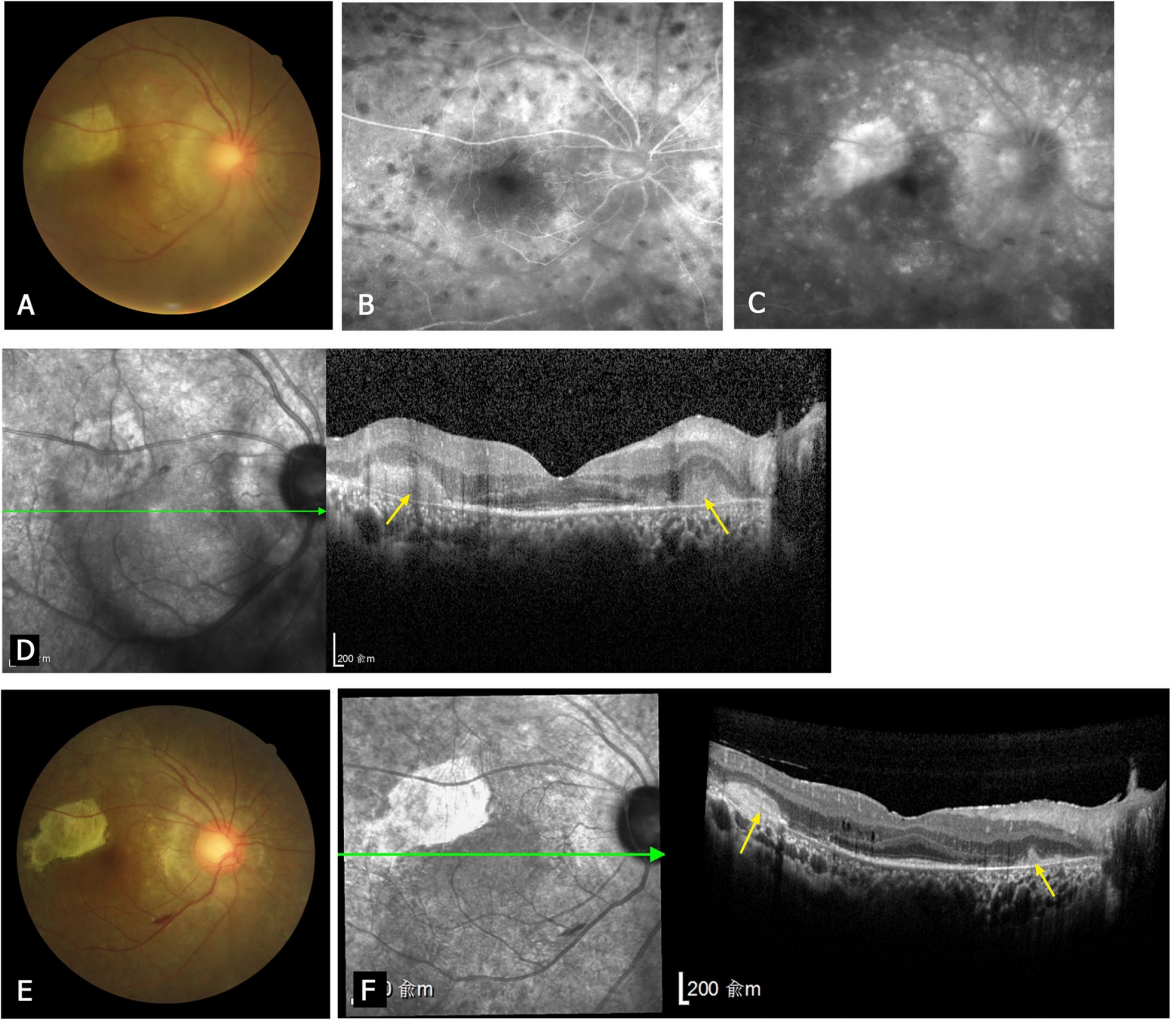
A 44-year-old patient with posterior sympathetic ophthalmia (PSO) accompanied by multifocal choroiditis. Fundus photography, fundus fluorescein angiography (FFA) and optical coherence tomography (OCT) images of the right eye (sympathizing eye). **a-d** Acute stage. **a** The fundus photograph shows yellow-white infiltrations in the macular and peripapillary areas that are accompanied by vitritis. **b** Early phase FFA shows choroidal hypofluorescent spots. **c** Late phase FFA shows fluorescence staining of the inflammatory lesions. **d** The OCT image shows inflammatory lesions on the retinal pigment epithelium (RPE; arrows) that protrude into the outer layer of the retina. The initial Snellen visual acuity was 20/400. **e, f** Fifteen months after treatment. **f** The OCT image shows shrinkage and hyperreflective fibrosis of the inflammatory lesions (arrows). The OCT image shows extensive loss of the external limiting membrane and ellipsoid zone, together with extensive atrophy of the RPE. The final Snellen visual acuity was maintained at 20/400

The inciting events, clinical manifestations, and treatments of the 48 patients with PSO are described in Additional file 1: Table S1. PSO could be classified into two types according to whether the fundus exhibited SRD or MFC. Images for two representative patients with SRD are shown in Figs. 1 and 2, and images for a representative patient with MFC are shown in Fig. 3. There were 41 patients (85.4%) with SRD (SRD group) and 7 patients (14.6%) with MFC (MFC group). Of the 41 patients with SRD, three had optic disc edema. MFC was characterized by multiple yellow–white inflammatory infiltrations of a variable number that were distributed from the posterior pole to the peripheral part of the fundus. The number of inflammatory lesions ranged from a few to more than 20. All of 7 patients with MFC had mild to moderate vitritis. FFA was performed in 12 patients with SRD and 3 patients with MFC. FFA of the patients with SRD revealed hyperfluorescent pinpoint leaks at the level of the RPE. FFA of the patients with MFC revealed choroidal hypofluorescent spots in early phase, and fluorescence staining of the inflammatory lesions in late phase.

All 48 patients received oral systemic corticosteroids. Four patients with SRD (9.8%) and 3 patients with MFC (42.9%) were also treated with cyclosporine. At the last follow-up, sunset-glow fundus was found in 18 cases (43.9%) of SRD group and in 2 cases (28.6%) of MFC group. There was no statistical difference in the frequency of sunset-glow fundus between the two groups (*P* = 0.683).

As indicated in Table 3, there were no significant differences in the sex, age, and inciting event between the SRD and MFC groups. However, the latent period was significantly longer in the MFC group (*P* = 0.0016). Table 4 compares the initial visual acuity and final visual acuity of the SRD and MFC groups. Visual acuity significantly improved in the SRD group (*P* < 0.0001) but significantly worsened in the MFC group (*P* = 0.0220). The final visual acuity was significantly worse in the MFC group than in the SRD group (*P* = 0.0001).

**Table 3:** Sex, age, latent period, and inciting event of patients with posterior sympathetic ophthalmia according to the presence of serous retinal detachment or multifocal choroiditis

Group	Sex (M/F)	Age (years), mean ± SD (median)	Latent period			Inciting event	
			< 3 months	3 months to 1 year	> 1 year	Trauma	Surgery
SRD group (*n* = 41)	35/6	44.9 ± 15.9 (45)	21	10	10	34	7
MFC group (*n* = 7)	7/0	51.6 ± 8.3 (51)	0	1	6	5	2
*P*-value	0.573	0.2925	0.002	0.601			

*M* male, *F* female, SD standard deviation, *SRD* serous retinal detachment, *MFC* multifocal choroiditis

**Table 4:** Initial and final visual acuity of patients with posterior sympathetic ophthalmia according to the presence of serous retinal detachment or multifocal choroiditis

	SRD group (*n* = 41)	MFC group (*n* = 7)	*P *value
Initial VA (LogMAR) mean ± SD (range)	0.76 ± 0.54 (2.0 to 0.0)	0.90 ± 0.36 (1.3 to 0.4)	0.2834
Final VA (LogMAR) mean ± SD (range)	0.27 ± 0.41 (2.0 to − 0.1)	1.49 ± 0.51 (2.0 to 0.8)	0.0001
*P*-value	< 0.0001	0.0220	

*SRD* serous retinal detachment, *MFC* multifocal choroiditis, *VA* visual acuity, *LogMAR* logarithms of the minimum angle of resolution

In the SRD group, the final visual acuity was obviously correlated with the initial visual acuity (*r* = 0.7105, *P* < 0.0001) and mildly correlated with age (*r* = 0.4556, *P* = 0.0028). In the MFC group, the final visual acuity was not correlated with the initial visual acuity or age (both *P* > 0.05).

### OCT features of PSO accompanied by SRD or MFC

The OCT images of patients with acute SRD revealed that the mean height of retinal detachment in the fovea was 528.8 ± 437.5 μm (range 88–1543 μm, median 415 μm). Among 41 patients with SRD, 26 (63.4%) had subretinal exudations, 14 (34.1%) had a membranous structure, and 24 (58.5%) had undulations of the RPE layer. The subretinal exudations always appeared as reflective amorphous materials in the subretinal space. The membranous structure appeared as a thin reflective layer on the surface of RPE, and/or septa crossing the retinal detachment. At the last follow-up after treatment, the OCT images showed that the retina had reattached and the subretinal fluid and exudations had dissolved in all 41 patients. Furthermore, the RPE layer had flattened. The band structures of the outer retina, including the external limiting membrane (ELM) and ellipsoid zone (EZ), were restored in 38 patients (92.7%). Only three patients (7.3%) had loss of ELM and EZ. The correlation between retinal detachment height and initial or final visual acuity was analyzed. The retinal detachment height was mildly correlated with the initial visual acuity (*r* = 0.4554, *P* = 0.0028), but not correlated with the final visual acuity (*P* > 0.05).

The OCT images of patients with acute MFC revealed inflammatory lesions on the RPE layer that protruded into the outer layer of the retina. At the last follow-up after treatment, the inflammatory lesions had shrunk and revealed fibrosis with increased reflectivity. All seven patients had loss of the band structures of the outer retina (i.e., ELM and EZ) and atrophy of the RPE.

## Discussion

In this retrospective study, we evaluated the clinical classification, long-term visual prognosis, and OCT features of 48 patients with PSO.

In this cohort, open globe injury was the most common inciting event followed by intraocular surgery. Vitrectomy was the most common type of intraocular surgery that resulted in PSO, consistent with previous reports [12, 13]. The latent period of PSO was similar between the trauma and surgery groups, and the type of inciting event was not significantly associated with the visual prognosis.

The patients in this cohort could be classified into two types of PSO. The first type, the most common, was characterized by SRD. The fundus manifestation of this type of PSO is similar to that of Vogt–Koyanagi–Harada Disease. Diffuse choroiditis could cause choroidal edema and leakage, leading to SRD. The second type, which was characterized by MFC, was less common. The prior knowledge of the clinical and OCT characteristics of PSO was mostly derived from studies of patients with PSO associated with SRD. Few reports have described that PSO can also be manifested by MFC. Pollack et al. [4] reported MFC in two of eight patients who developed PSO after vitrectomy. However, the authors did not describe these cases in detail or perform OCT. In our study, there were seven patients with PSO accompanied by MFC, and we performed OCT in all seven patients. MFC is a descriptive diagnosis based on the fundus manifestations, with a variety of causes, including ocular tuberculosis [14] and systemic autoimmune diseases such as sarcoidosis [15]. In our study, we excluded ocular infectious diseases and systemic autoimmune diseases as possible diagnoses in all seven patients. Idiopathic MFC predominantly affects young, healthy women [16]. Unlike idiopathic MFC, the seven patients with PSO accompanied by MFC were males with a history of trauma or intraocular surgery of one eye. Two of the patients with PSO accompanied by MFC had sunset-glow fundus in the late stage of the disease.

Sympathetic ophthalmia is an autoimmune response to the exposure of ocular tissue antigens, and is associated with genetic susceptibility. Although PSO and Vogt–Koyanagi–Harada disease have similar human leukocyte antigen genotypes [17, 18], Deng et al. [19] suggested that these two diseases have different single nucleotide polymorphism susceptibility loci. In this study, we found that the latent period was significantly longer in patients with MFC than in patients with SRD. Because the sensitization latency differs between types of hypersensitivity reactions [20], we speculate that the significant difference in the latent period between the two types of PSO indicates that they involve distinct immune mechanisms. The differences in the immunogenetic backgrounds may lead to different immune mechanisms between the two types of PSO, a possibility that needs further study.

We found that the two types of PSO had completely different OCT manifestations. The predominant OCT features in patients with acute SRD were subretinal fluid and exudations, and the membranous structure was also found in some of the patients. The membranous structure may also appear in patients with Vogt–Koyanagi–Harada disease, and it seems to be composed of inflammatory substances and photoreceptor outer segments [21]. The main OCT features of acute MFC were inflammatory lesions on the RPE layer. After treatment, the ELM and EZ bands were restored in most patients with SRD, but they remained disrupted in patients with MFC. The integrity of the band structures of the outer retina (especially EZ) was reported to be significantly associated with good visual function [22, 23].

We found that the two types of PSO had different visual prognosis. The MFC type PSO has more serious retinal destruction and worse visual prognosis. Therefore, MFC type PSO needs to try more aggressive treatment and explore more effective immunosuppressants. Additionally, we found that the final visual acuity was correlated with the initial visual acuity in patients with SRD. This suggests that early treatment may improve the visual prognosis in patients with PSO accompanied by SRD.

In conclusion, we found that there are two main clinical types of PSO, based on the presence of either SRD or MFC. The visual prognosis differed significantly between these two types of PSO. OCT imaging is particularly helpful for the clinical classification and monitoring of retinal changes after treatment in patients with PSO.

## Supplementary Information

**Additional file 1.** The inciting events, clinical manifestations and treatments of the 48 patients with posterior sympathetic ophthalmia.

## Data Availability

The data used to support the findings of this study are available from the corresponding author upon request.

## Abbreviations

ELM: External limiting membrane
EZ: Ellipsoid zone
FFA: Fundus fluorescein angiography
LogMAR: Logarithms of the minimum angle of resolution
MFC: Multifocal choroiditis
OCT: Optical coherence tomography
PSO: Posterior sympathetic ophthalmia
RPE: Retinal pigment epithelium
SRD: Serous retinal detachment
VA: Visual acuity
