# Supplementary material for: Clinical classification, visual outcomes, and optical coherence tomographic features of 48 patients with posterior sympathetic ophthalmia
Source: Orphanet J Rare Dis. 2022 Mar 4;17:103. doi: 10.1186/s13023-022-02258-0 (PMC8895912; doi:10.1186/s13023-022-02258-0)
Supplement: Supplementary file 1 — Additional file 1. The inciting events, clinical manifestations and treatments of the 48 patients with posterior sympathetic ophthalmia. [file 13023_2022_2258_MOESM1_ESM.docx]

**Supplemental Table 1.** The inciting events, clinical manifestations and treatments of the 48 patients with posterior sympathetic ophthalmia

| Case No. | Sex | Age | Inciting event | Anterior chamber inflammation | Vitritis | Fundus manifestation | Treatment |
| --- | --- | --- | --- | --- | --- | --- | --- |
| 1 | M | 29 | Trauma | Mild | Mild | SRD | Steroids |
| 2 | M | 58 | Trauma | Mild | Mild | SRD | Steroids |
| 3 | M | 73 | Trauma | Mild | Mild | SRD | Steroids |
| 4 | M | 61 | Trauma | Mild | Mild | SRD | Steroids |
| 5 | M | 37 | Trauma | Mild | Mild | SRD | Steroids, cyclosporine |
| 6 | M | 54 | Trauma | Mild | Mild | SRD | Steroids |
| 7 | F | 46 | Trauma | Negative | Mild | SRD | Steroids |
| 8 | M | 51 | Trauma | Mild | Mild | SRD | Steroids, cyclosporine |
| 9 | M | 51 | Trauma | Negative | Mild | SRD | Steroids |
| 10 | M | 35 | Trauma | Negative | Negative | SRD | Steroids |
| 11 | M | 45 | Trauma | Mild | Mild | SRD | Steroids |
| 12 | M | 70 | Trauma | Mild | Mild | SRD | Steroids |
| 13 | M | 20 | Trauma | Mild | Mild | SRD | Steroids |
| 14 | M | 24 | Trauma | Mild | Mild | SRD | Steroids |
| 15 | F | 27 | Trauma | Mild | Mild | SRD | Steroids |
| 16 | F | 40 | Trauma | Mild | Mild | SRD | Steroids |
| 17 | M | 53 | Trauma | Mild | Mild | SRD, ODE | Steroids |
| 18 | M | 57 | Trauma | Mild | Mild | SRD | Steroids |
| 19 | M | 67 | Trauma | Mild | Mild | SRD | Steroids |
| 20 | M | 65 | Trauma | Negative | Negative | SRD | Steroids |
| 21 | M | 51 | Trauma | Negative | Negative | SRD | Steroids |
| 22 | M | 66 | Trauma | Mild | Mild | SRD | Steroids |
| 23 | M | 53 | Trauma | Mild | Mild | SRD | Steroids |
| 24 | M | 41 | Trauma | Mild | Mild | SRD | Steroids |
| 25 | M | 63 | Trauma | Mild | Mild | SRD | Steroids |
| 26 | M | 67 | Trauma | Mild | Mild | SRD | Steroids |
| 27 | M | 10 | Trauma | Mild | Mild | SRD | Steroids |
| 28 | M | 65 | Trauma | Mild | Mild | SRD | Steroids |
| 29 | M | 24 | Trauma | Mild | Mild | SRD | Steroids |
| 30 | F | 23 | Trauma | Mild | Mild | SRD | Steroids |
| 31 | F | 39 | Trauma | Mild | Moderate | SRD | Steroids, cyclosporine |
| 32 | M | 37 | Trauma | Mild | Mild | SRD | Steroids |
| 33 | M | 30 | Trauma | Negative | Negative | SRD, ODE | Steroids |
| 34 | M | 31 | Trauma | Negative | Negative | SRD | Steroids |
| 35 | M | 51 | Vitrectomy | Mild | Mild | SRD | Steroids |
| 36 | M | 29 | Vitrectomy | Mild | Moderate | SRD | Steroids, cyclosporine |
| 37 | M | 48 | Vitrectomy | Mild | Mild | SRD | Steroids |
| 38 | F | 45 | Vitrectomy | Mild | Mild | SRD | Steroids |
| 39 | M | 31 | Vitrectomy | Mild | Mild | SRD | Steroids |
| 40 | M | 27 | Vitrectomy | Negative | Negative | SRD, ODE | Steroids |
| 41 | M | 45 | Anti-glaucoma surgery | Mild | Mild | SRD | Steroids |
| 42 | M | 51 | Trauma | Mild | Moderate | MFC | Steroids, cyclosporine |
| 43 | M | 44 | Trauma | Mild | Moderate | MFC | Steroids, cyclosporine |
| 44 | M | 49 | Trauma | Mild | Mild | MFC | Steroids |
| 45 | M | 43 | Trauma | Mild | Mild | MFC | Steroids |
| 46 | M | 68 | Trauma | Mild | Moderate | MFC | Steroids |
| 47 | M | 52 | Vitrectomy | Mild | Moderate | MFC | Steroids, cyclosporine |
| 48 | M | 54 | Cataract extraction | Mild | Moderate | MFC | Steroids |

M male, F female, SRD serous retinal detachment, MFC multifocal choroiditis, ODE optic disc edema
